# Odour Profiling for Animal Management and Behavioural Manipulation: An Overview of the Tools and Applications

**DOI:** 10.1007/s10886-026-01770-y

**Published:** 2026-09-29

**Authors:** Ashlyn Austin, Adrian D. Manning, Todd A. Gillam, Anton Blencowe

**Affiliations:** 1 Applied Chemistry and Translational Biomaterials (ACTB) Group, Centre for Pharmaceutical Innovation (CPI), School of Pharmacy and Biomedical Sciences, College of Health, Adelaide University, Adelaide, SA 5000 Australia; 2 Fenner School of Environment and Society, The Australian National University, Canberra, ACT 2601 Australia; 3 School of Mathematics, Statistics, Chemistry and Physics, College of Science, Technology, Engineering and Mathematics, Murdoch University, Perth , WA 6150 Australia

**Keywords:** Odour profiling, Behavioural manipulation, Volatile organic compounds, Analysis techniques, Sampling techniques, Ethology

## Abstract

Olfaction plays a prominent role in the evolution of animal behaviour, facilitating both intraspecies and interspecies communication, and predator-prey interactions. Volatile organic compounds (VOCs) underpin these odours, and their analysis is therefore central to characterising and replicating cues such as pheromones, allelochemicals, and other released signals used to attract, deter, or condition animal responses. Thus, this review aims to introduce the reader to the applications of animal odours in animal management and behavioural control, and to provide a critical, practice-oriented guide to selecting and combining VOC sampling and analytical techniques for animal odour studies. Animal odour profiling typically involves three steps: (1) sampling, (2) transfer to the instrument, and (3) detection/identification of VOCs. Gas chromatography–mass spectrometry (GC–MS) is the gold standard for VOC analysis, and is often paired with headspace techniques, including static headspace, dynamic headspace, and solid-phase microextraction (SPME) to facilitate sampling and transfer of VOCs. In addition, liquid and distillation-based extraction methods are discussed, which remain important for capturing less volatile or matrix-bound components in animal odour and semiochemical research. On the analytical side we discuss GC–MS alongside emerging and complementary techniques, including proton transfer reaction–mass spectrometry (PTR–MS), selected ion flow tube–mass spectrometry (SIFT–MS), gas chromatography–olfactometry (GC–O), and electronic noses, emphasising their respective strengths and limitations for qualitative and quantitative VOC analysis. Collectively, the literature shows that no single sampling or analytical approach is universally optimal. Effective animal odour profiling therefore requires matching extraction and detection strategies to the volatility, stability, and behavioural relevance of target VOCs. Careful consideration should also be given to how these methodological choices influence the odour profiles used in ecological, behavioural, and applied management contexts.

## Introduction

The analysis of volatile organic compounds (VOCs) is essential to the characterisation and replication of odours from their constituent molecular components. Olfaction, the sense of smell, has played a prominent role in the evolution of animal behaviour, by facilitating predator-prey interactions, interspecies and intraspecies communication, and behavioural development in animals (Apfelbach et al. 2005; Banks et al. 2014; Nielsen et al. 2015). Odours, such as pheromones or allelochemicals, form vital signatures for social communication (Deshpande et al. 2018). More specifically, pheromones are used in communication between individuals of the same species, whilst allelochemicals are used for interspecies communication (Kost 2008). Research shows that particular VOCs may be species-specific, which is essential to the olfactory recognition of different species (Deshpande et al. 2018; Holderman et al. 2017). These odours persist within the environment, to be exploited by predators to detect prey (Banks et al. 2014; Hughes et al. 2012). For instance, VOCs within urine and faecal scent marks persist for many days or weeks and are used by both predators and prey to strategically avoid or enhance detection (Banks et al. 2016; Hughes et al. 2012).

Olfaction plays a significant role in predator-prey relationships. Canids, such as foxes (*Vulpes vulpes*), have a keen sense of smell, essential to the identification and tracking of the prey they hunt and the food they scavenge (Banks et al. 2014). There is evidence that olfaction plays a significant role in the hunting behaviours of foxes, as noted by Hughes et al., whereby foxes frequently visited scent marks of house mice (*Mus musculus*) (Hughes et al. 2010). However, there is also evidence that foxes may be especially attracted to novel prey odours (neophilia), as demonstrated by Bytheway et al. who assessed fox attraction was greater towards the novel long-nose bandicoot (*Perameles nasuta*) than the co-evolved black rat (*Rattus rattus*) (Bytheway et al. 2016). This is an alarming finding in the case of foxes as introduced predators, because they can potentially adapt to hunting and predating previously unfamiliar native fauna.

The function of animal odours as lures or chemical attractants and deterrents can be exploited to influence animal behaviour. This can be achieved using natural odours obtained from bodily excretions, or through synthetic mimics which replicate the animal odour. Animal odours from urine, faeces, and bedding, have all been successfully implemented as lures for trapping in instances where food lures have been unsuccessful (Murphy et al. 2022).

Individual animal VOCs are generally simple organic molecules, typically aliphatic, aldehyde, ketone, alcohol, amine, carboxylic acid and sulphur containing compounds (Holderman et al. 2017). However, whole animal odours are complex mixtures of VOCs, each of which can have varying levels of volatility, impacting odour longevity (Doty 1986). Whilst some pheromone compounds have been elucidated, little is known about which allelochemical compounds within particular animal odour profiles are key to the elicitation of odour responses (Banks et al. 2014; Hughes et al. 2012). However, allelochemicals are usually broken down into who they benefit; kairomones that benefit the receiver and allomones that benefit the emitter (Brown et al. 1970). Understanding the chemical composition of these odours by VOC analysis can therefore assist in the design of practical and effective lures, repellents, and other behaviour manipulation tools, produced from key VOCs. These can then be used to influence animal behaviour, such as reducing or enhancing a particular response.

This review first outlines the importance of animal odour profiling by discussing its current and emerging applications. Subsequently, we provide a critical overview of the primary analytical techniques and approaches used to profile animal odours, as well as odours more generally. The particular advantages and limitations of the various approaches to animal VOC profiling are discussed, with special consideration given to whether the objective is to detect a known component or to elucidate the composition of an unknown mixture. Key examples of animal odour characterisation are presented, highlighting important considerations relevant to the selection and application of particular analytical techniques.

## The Significance of Animal Odour Determination to Applied Zoology and Ecology

Odours comprise the important signals that many animals are dependent upon for communication and situational awareness; as such, their behaviours may also be manipulated using these odours (Deshpande et al. 2018). Identification of key VOCs is critical to understanding the odour-based behaviours of animals. Animal olfactory responses can be manipulated to influence animal behaviour, particularly if VOCs that are essential to scent cues are identified for use. Animal odour cues may be either synthetically replicated or naturally sourced.

### Behavioural Manipulation

Comprising both volatile and non-volatile compounds, pheromones are the chemical cues that influence same-species communication, impacting both social and reproductive behaviour (Stowers and Marton 2005). Pheromones interact with chemoreceptive organs such as the vomeronasal system or main olfactory epithelium, resulting in innate behaviours that do not require prior experience or exposure, such as suckling and aggression (Bargmann 1997; Stowers and Marton 2005). Pheromones can be broken down into subclasses including, but not limited to, alarm, trail, territorial, and sex pheromones (Bargmann 1997; Liberles 2014; Stowers and Marton 2005). Whilst select pheromonal compounds have been identified, many remain chemically uncharacterised (Frank et al. 2010). Some studies have identified compounds with potential pheromonal activity in male mouse urine, resulting in oestrus induction, increased male aggression, female attraction and puberty acceleration or delay (Cavaliere et al. 2020; Dulac and Torello 2003; Jemiolo et al. 1986; Novotny et al. 1985, 1999). Additionally, mouse tears were found to contain pheromones that had differing effects depending on their source; female tears displayed anti-aggressive effects, male tears resulted in pregnancy blocks on females, and pup tears inhibited male mating behaviour and sexual rejection in females (Cavaliere et al. 2020).

Use of pheromones in behavioural manipulation has been most prominent in the veterinary field to reduce fear and anxiety in dogs (*Canis lupus familiaris*) and cats (*Felis catus*), as well as to reduce urine marking in domestic animals (Pageat and Gaultier 2003; Tod et al. 2005). Dog appeasing pheromone (DAP) and feline facial pheromone (FFP), sold under the brand names Adaptil^®^ and Feliway^®^_,_ respectively, were developed as synthetic analogues of pheromones to be used to reduce undesirable behaviours and stress/fear (Pageat and Gaultier 2003). FFP mimics the natural markings that result from cats rubbing their faces on domestic objects, and was developed as a treatment for anxiety related behaviours, such as non-sexual urine spraying (Frank et al. 1999; Gunn-Moore and Cameron 2004). A study investigating the use of FFP as an anxiolytic in hospitalised cats saw increased behaviours such as grooming, eating and facial rubbing when cats were exposed to FFP in their cages, indicating the cats were more at ease (Griffith et al. 2000). Similar studies have been conducted in dogs using DAP, which mimics the pheromone produced by lactating females post-birth (Pageat and Gaultier 2003; Tod et al. 2005). Landsberg et al. found that dogs fitted with DAP collars displayed significantly reduced fear/anxiety when exposed to thunderstorm simulations, as compared to dogs with placebo collars (Landsberg et al. 2015).

Odours can also be employed to modify animal behaviour through associative learning processes such as conditioned odour aversion (COA) or conditioned taste aversion (CTA) (Cowan et al. 2000; Manning et al. 2021). In COA, an odour becomes aversion after being paired with a negative experience, whereas CTA typically arises when a taste cue is paired with a negative post-ingestive experience (e.g., non-lethal illness), leading animals to subsequently avoid stimuli associated with that cue. From an evolutionary perspective, CTA is considered an adaptive mechanism that reduces the likelihood of animals re-consuming toxic substances (Foy and Foy 2009). While CTA is often discussed in the context of taste, olfactory cues accompanying a food can also act as a conditioned cue, enabling avoidance responses to be elicited by similarly scented foods, baits, or other targets.

The CTA strategy has been explored in a range of ecological, conservation and pest management contexts. For example, CTA has been used in predator management studies involving coyotes (*Canis latrans*) (Gustavson et al. 1974), dingoes (*Canis lupus dingo*), wild dogs (*Canis hallstromi*) (Gustavson et al. 1983), and foxes (Andrewartha et al. 2023), as well as to train native predators to avoid toxic or invasive prey (Jolly et al. 2017). These studies demonstrate the potential of associative aversion learning as a behavioural-management tool, although they do not necessarily isolate the specific contribution of olfactory cues. The effectiveness of conditioned aversion can also depend on factors such as prior experience, social context, and whether conditioning is conducted in captive or free-ranging animals (Gustavson et al. 1974, 1983; Ward-Fear et al. 2017). Importantly, recent studies highlight that the presence and nature of the odour cue significantly affect the strength and persistence of the learned aversion. While some work has used non-ecologically relevant odours such as vanilla, these still enhance aversion retention, likely through taste-potentiated odour aversion mechanisms (Tobajas et al. 2021). Tobajas et al. showed that when an odour cue was incorporated into a captive CTA regime, aversive behaviour was retained for 8–11 months (Tobajas et al. 2019). Although most studies have been conducted with captive predators, Andrewartha et al. demonstrated that wild foxes can be conditioned using fried chicken baits containing an aversive agent, with the effect persisting for at least 70 days, until the end of the study (Andrewartha et al. 2023). Together, these findings emphasise that odours are not merely auxiliary cues but can play a central role in the durability and transferability of CTA-based behavioural modification.

### Chemical Attractants and Deterrents

Lures and attractants are beneficial where trapping practices are implemented for predator control and can allow for the relocation of native predators, rather than culling. A study investigating the use of bedding odours as lures for stoats (*Mustela erminea*) demonstrated that the bedding of oestrus females could be used as an effective trap lure for both male and female stoats (Murphy et al. 2022). Captive studies found that both male and female stoats spent significantly more time investigating the treatment lures than a control lure, both in and out of breeding season. The bedding lures were also seen to be equally as effective, if not more so, than dried rabbit (*Oryctolagus cuniculus*) or chicken (*Gallus gallus domesticus*) egg lures.

Alternatively, Cork and Hall demonstrated that a synthetic odour can be used to successfully mimic live sentinel animals in bait station monitoring (Cork and Hall 2007). This study compared the behaviour of screw-worm flies (*Cochliomyia hominivorax*) around both live sheep (*Ovis aries*) baits and variations of a synthetic wound fluid. A major finding of this study was that the synthetic wound fluid could be used to elicit the same behavioural responses as natural wound fluid, with the most notable differences observed in the male flies. Johansson et al. also successfully demonstrated that a synthetic odour can be used to mimic that of a live insect, when training sniffer dogs to detect bark beetle (*Ips typographus*)-infested trees using synthetic pheromones (semiochemicals) (Johansson et al. 2019). By training the dogs with four different synthetic pheromones the dogs were capable of detecting bark-beetle infested trees from up to 150 m away, which is estimated to be further away than what bark beetles respond to, and up to 100 times further away than humans can detect (Johansson et al. 2019; Schlyter et al. 1989). This study highlights that canines can reliably respond to relatively small amounts of the odour across substantive distances.

Odours can also be used as deterrents. Deterrent signals can be beneficial to prey when avoiding predators. Various studies have demonstrated the avoidance of predator odours by prey animals, as well as other behavioural changes such as reduced non-defensive behaviours (e.g., grooming, foraging and reproduction) (Apfelbach et al. 2005; Dickman and Doncaster 1984; Hubbard et al. 2004; Jolly et al. 2017). For instance, rodents (*Rodentia*) readily avoid traps tainted with fox faecal odours, but not rodent faecal odour (Dickman and Doncaster 1984). Conversely, rodents were not as avoidant of traps tainted with badger (*Meles meles*) odour. As small mammals only comprise a minor fraction of a badger’s diet, and badgers only defecate at latrines, it is likely that rodents have less of an opportunity to develop an aversion to their odour.

Although some studies show that prey animals actively avoid odours from predator species, others report more cautious but relatively weak avoidance responses (Garvey et al. 2016). Several explanations have been proposed for this pattern, including the need for prior experience, inability to discriminate predator odours, variation in the age or amount of odour, and the potential fitness costs of avoiding a possible food source (Apfelbach et al. 2005; Harrington et al. 2009; Sih et al. 2010; Ward et al. 1997). It is also likely that since there were no auditory or visual confirmation of a threat upon investigation, that animals tend to cease their cautious behaviour and do not repeat that behaviour on future visits/interactions (Garvey et al. 2016). Garvey et al. found that stoats cautiously approached and scanned foraging units containing both sympatric and non-sympatric predator odours. However, when no visual or auditory threat was detected, the stoats ceased these behaviours and did not display any cautious behaviour on subsequent visits (Garvey et al. 2016).

Additionally, odour can be used for camouflage or misinformation to protect areas or species of conservation interest (Norbury et al. 2021; Price et al. 2022). Because odours are often the first cue available for detecting a food source, prior to sight or sound, they represent an effective target for misinformation strategies that deter predators or foragers before visual or auditory confirmation of a food source (Price et al. 2022). For example, Norbury et al. demonstrated that repeated exposure of invasive mammals in New Zealand to unrewarding bird odours, scent cues that are not followed by access to prey or any food reward, prior to the arrival and nesting of native shorebirds increased hatching success. The invasive mammals included the following species: ferrets (*Mustela putorius furo*), cats, and European hedgehogs (*Erinaceus europaeus occidentalis*), and the shorebirds included Plover (*Charadrius bicinctus*), wrybill (*Anarhynchus frontalis*), and South Island pied oystercatcher (*Haematopus finschi*). Depending on the bird species, the probability of successful hatching more than doubled or even tripled (Norbury et al. 2021).

Olfactory misinformation/camouflage has also been used to protect plant species from foraging macropods. This approach involves placing artificial odours of undesirable plants around seedlings of palatable species to provide a browsing refuge and increase regeneration and survival of seedlings (Finnerty et al. 2024). Alternatively, dispersing the odour emitted by the species of interest can make food sources more difficult for foragers to find, ultimately reducing their interest in that odour (Parker et al. 2023). Parker et al. showed that camouflaging an area with wheat germ odour resulted in 63% fewer diggings by mice. Similar results were obtained using unrewarded pre-exposure to the odour prior to wheat seed sowing, which reduced mouse diggings by 74%. These findings suggested that both approaches could contribute to non-lethal pest control strategies (Parker et al. 2023).

It is evident that odours and olfaction play essential roles in predator-prey interactions, interspecies communication, and behavioural development in animals (Apfelbach et al. 2005; Banks et al. 2014; Nielsen et al. 2015). Odours are important in situations where visual or auditory signals are limited, as they can disperse through space and persist in the environment for long periods of time (Doty 1986). Consequently, odours can be used and manipulated for applications in animal management and behavioural control. However, achieving this requires a detailed understanding of the VOCs that constitute these odours, as well as effective methods for their sampling and characterisation.

## Analytical Approaches for the Characterisation of Odours

Analysis of animal VOCs typically consists of three key stages; (1) sampling, (2) transfer of the sample to an analytical instrument, and (3) detection and identification of compounds (Posudin 2014). Sampling of VOCs can be conducted using a range of techniques including headspace extraction, liquid extraction and hydrodistillation, which to some extent dictate how the sample extracts are transferred and analysed. The selection of an appropriate analytical technique for VOC analysis depends on the sample type and the analytical objective, but in the majority of cases involving animal odour profiling, gas chromatography–mass spectrometry (GC–MS) remains the method of choice due to its versatility, accessibility and relatively low cost. Nevertheless, when analysing known VOCs, other techniques such as GC coupled with flame ionisation or photoionisation detectors are also commonly employed. In addition, other complementary techniques have emerged including gas chromatography–olfactory (GC–O), proton transfer reaction–mass spectrometry (PTR–MS), selected ion flow tube–mass spectrometry (SIFT–MS) and the electronic nose; while the use of these techniques for animal odour profiling has been limited to date, they remain important for VOC analysis.

### Sampling Techniques

The choice of VOC sampling method typically depends on the sample matrix and nature of the VOCs, whether analysis is online or offline, and whether the goal is qualitative profiling, quantification, storage, or field deployment (Haug et al. 2024). Given that VOCs are typically defined as compounds that have transitioned from a sample matrix into the surrounding air, sampling usually proceeds in the gas phase using headspace extraction techniques, which are particularly prominent in animal odour profiling where identification of specific VOCs or VOC profiles are the key objective. In comparison, liquid extraction and hydrodistillation are also used for the isolation of VOCs from specific samples, especially plants, water and soils, but neither is universal because recovery depends on matrix, solvent, temperature, and analyte volatility (Demeestere et al. 2007). While the use of liquid extraction and hydrodistillation can provide access to isolatable quantities of extracts, they are rarely used in animal odour profiling as they may underrepresent highly volatile compounds and the resulting extracts may not faithfully reproduce the gas-phase VOC profile (Table 1).

**Table 1:** Comparison of extraction techniques in terms of their applications, strengths, limitations and profile biases

Extraction technique	Basic principle	Application	Strengths	Limitations	Profile bias
Headspace extraction	Samples VOCs that partition into the gas phase above a solid or liquid matrix	Rapid profiling of VOCs from foods, plants, animals, consumer products, water, and biofluids	Minimal sample preparation, reduced matrix interference, automation potential, and often solvent-free	Strongly affected by temperature, equilibration, sorbent phase, and headspace conditions	Favours more volatile compounds; fibre/sorbent selectivity can skew composition
Liquid extraction	Transfers VOCs from aqueous or solid samples into an organic solvent	Water, soils, biological tissues, and matrix-associated VOCs poorly recovered by gas-phase methods	Can recover compounds retained in liquid or solid phases; extracts can be stored and reanalysed	Solvent choice drives selectivity; methods can be more laborious and solvent-consuming	Favours less-volatile or matrix-retained compounds relative to gas-phase only methods
Distillation-based extraction	Heats the sample with water or steam and recovers released volatile compounds by condensation	Essential oils and bulk recovery of volatile fractions from plant and other biological materials	Established, preparative, and useful for isolating volatile fractions for downstream analysis	Time-consuming, often requires more sample material, and can degrade thermolabile analytes	Favours compounds that withstand heating and distillation; thermal degradation or transformation can generate artefacts

#### Headspace Extraction Techniques

**Fig. 1:**
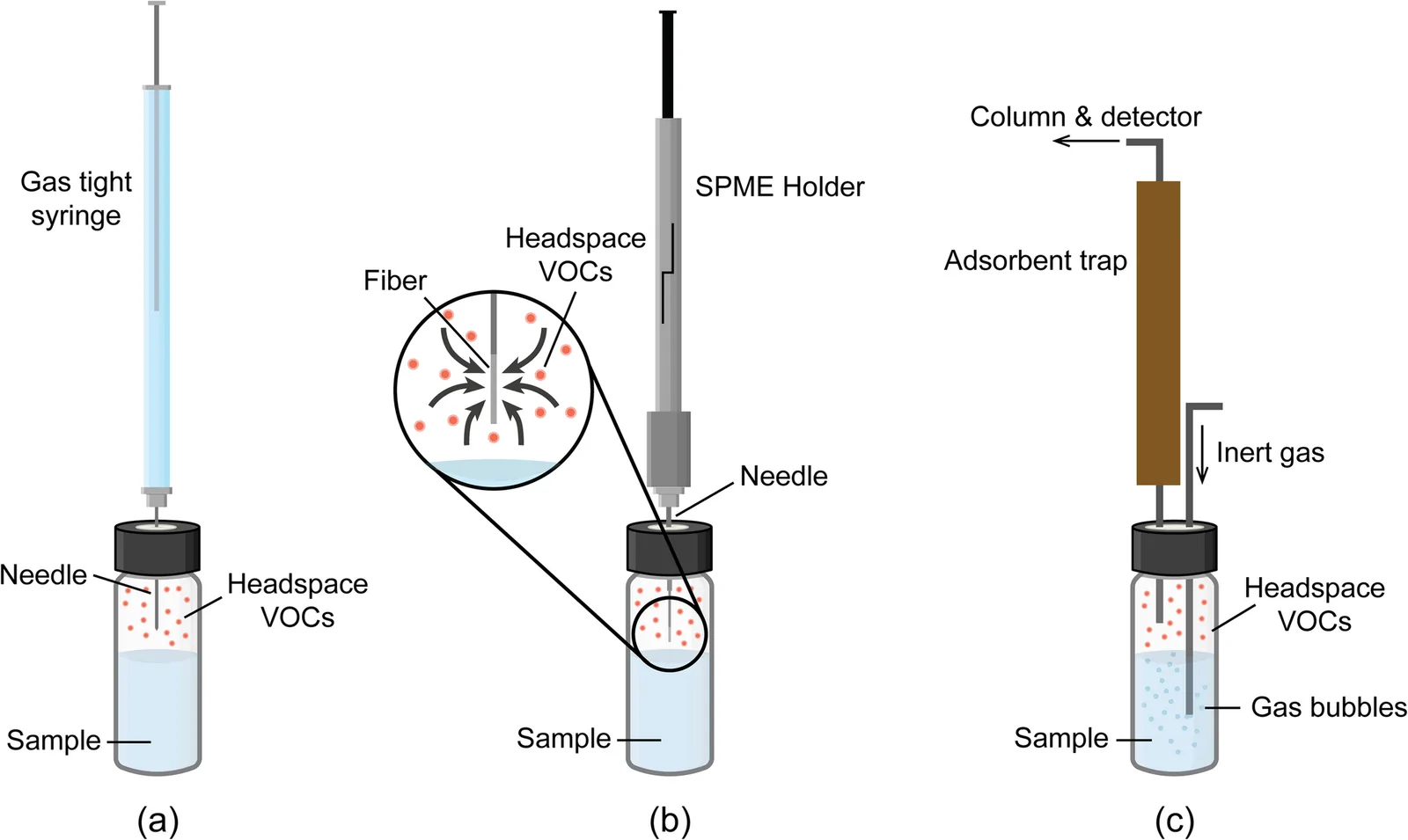
(**a**) static headspace extraction; (**b**) headspace solid-phase microextraction; (**c**) dynamic headspace (purge and trap)

Static headspace extraction (SHE) involves heating a sample in a sealed vial until the VOCs reach equilibrium between the gas phase and the liquid or solid sample (Fig. 1a) (Flórez Menéndez et al. 2004; Jeleń et al. 2017). Once equilibrium is established, an aliquot of the gas phase is transferred directly to the GC instrument using a gas tight syringe (Kremser et al. 2016). Solid-phase microextraction (SPME) is similar to SHE, except that SPME uses a fused silica fibre coated with a polymeric phase that combines sampling and sample concentration in a single step (Fig. 1b). Polymeric fibre coatings include polydimethylsiloxane, polyacrylates, and mixed phases. The selection of an appropriate fibre coating, or sorptive material, therefore depends on the types of compounds of interest (van Hout et al. 2003), and the extract composition can be dependent on the choice of sorptive material. SPME can be used for either headspace sampling, or direct adsorption from a liquid sample (Finnerty et al. 2022; Flórez Menéndez et al. 2004). Following adsorption of VOCs to the SPME fibre, the fibre is then inserted into a GC injector, where the compounds are thermally desorbed onto the column. Because VOCs are concentrated on the fibre, SPME provides greater sensitivity than standard SHE, and allows the analysis of very small sample amounts (Cavalli et al. 2003). Headspace SPME has also been further developed through the introduction of a sample enrichment probe (SEP), a high-capacity sampling technique (Burger et al. 2006). The main difference between SPME and SEP is the volume of the sorptive phase. SEP uses a larger volume of the sorptive phase, allowing greater adsorption of complex mixtures and improving detection sensitivity. Direct sampling of VOCs using headspace techniques allows solvent-free extraction, and in the case of SPME, high sensitivity for low boiling point compounds (Jeleń et al. 2017).

However, results are influenced by numerous variables including; temperature, samples and container size, ratio of sample to headspace volume, nature of the sample matrix, addition of matrix modifiers, and whether the sample is stirred during equilibration (Cadwallader 2007). This means that although these are relatively simple techniques, a small change in conditions can easily influence the results.

The main advantages of SHE and SPME are simple sample preparation, solvent free extraction, and the low risk of artifacts due to the closed system sampling (Cadwallader 2007). However, these methods also have limitations, including the inherent bias to low molecular weight and low boiling point compounds and difficulty in quantitative analysis beyond peak-area comparisons and relative ratios (Cadwallader 2007; Walker and Vaglio 2021). A further limitation of SHE is its low sensitivity due to the lack of a sample concentration step (Flórez Menéndez et al. 2004; Jeleń et al. 2017).

SPME techniques have been used to profile the odour of various species including, sheep and horses (*Equus caballus*). Sheep odours have been characterised using either wool or wool grease. Several studies have reported that wool grease contains VOCs such as aliphatics, aldehydes, ketones, alcohols, and carboxylic acids with carbon chain lengths ranging from five to ten (Burger et al. 2013; Lisovac and Shooter 2003). More specifically, Lisovac et al. found ethanol, acetone, pentanal, hexanal and various branched aliphatics in both wool and wool grease (Lisovac and Shooter 2003). Additionally, Burger et al. used SEP to demonstrate that twin lambs possess unique odour profiles that are more similar to each other than those of randomly selected non-twin lambs (Burger et al. 2011).

A study of the odour profiles of two horse breeds; Appaloosa (*n* = 6) and Quarter Horses (*n* = 17), found that all sampled individuals shared several common VOCs (Deshpande et al. 2018). Among the 187 VOCs identified, nonanal, 3,6-dimethoxy-9-(2-phenylethynyl)-fluoren-9-ol, and 1,2-dibromo-2-methyl-undecane were the only compounds present in all horse samples. The differences among individuals were primarily attributed to variation in compound concentrations rather than the presence or absence of specific compounds. Such variation may also influence the intermolecular interactions between those compounds, and consequently, their volatilisation (Deshpande et al. 2018).

Dynamic headspace (DHS), also known as purge and trap, is similar to SPME in that VOCs are collected on a sorbent material, using a solventless technique (Fig. 1c) (Bruno and Harries 2019). The main difference that DHS offers is in how VOCs are removed from the matrix or sample. In DHS, the sample is purged with an inert gas, such as nitrogen or helium, to extract VOCs (Abeel et al. 1994). The flowing gas carries the VOCs onto a sorbent material, such as sorbent polymers (e.g. Tenax^®^) or graphitised carbon black (e.g. Carbopack™) (Mallia et al. 2005; Vyviurska et al. 2021). The analytes can then be desorbed for subsequent analysis. DHS provides sensitivity comparable to that of SPME. However, artifacts may result from degradation of the sorbents (Marsili 1999). DHS is also less commonly used because it is generally more expensive than comparable techniques. On the other hand, an advantage of DHS techniques is the ability to sample whole animals, rather than smaller samples of fur or excretions. For example, Holderman et al. demonstrated direct sampling of domestic dogs (*n* = 4) using vacuum rather than gas flow, to extract VOCs that were subsequently concentrated on Tenax^®^ (Holderman et al. 2017). This study found 41 VOCs common to all dogs including aliphatics, ketones, aldehydes, alcohols, and carboxylic acids. Other studies have reported the presence of several aliphatics with 11–21 carbons (de Oliveira et al. 2008). Both studies found that changes in VOC profiles could be used to detect parasitic infections (de Oliveira et al. 2008; Holderman et al. 2017).

#### Distillation

An alternative to headspace techniques is distillation-based extraction, such as hydrodistillation, which has previously been used for the extraction of volatiles from wool (Tsikolia et al. 2022). Hydrodistillation, commonly used for oil extraction, involves boiling wool in water to release oils or VOCs, and collecting the resulting vapour (Hasbay and Galanakis 2018). The condensed water vapour can then be extracted using an organic solvent to isolate the VOCs prior to liquid injection into a GC–MS. As previously mentioned, extract composition can depend on the extraction technique, including choice of sorptive material in SPME and DHS. The type of extraction, headspace or distillation, can also influence extract composition. Hydrodistillation can extract large quantities of semi-volatile compounds that do not necessarily contribute to the odour, and the high temperatures involved can induce thermal degradation, leading to detection of artifactual compounds (Tsikolia et al. 2022). Additionally, hydrodistillation typically requires much larger sample sizes compared to headspace techniques, which can be impractical depending on the animal odour being investigated.

Tsikolia et al. compared hydrodistillation, DHS, and headspace SPME with three different fibre coatings to analyse VOCs in stored sheep wool (Tsikolia et al. 2022). The study revealed significant differences in the VOCs detected by each method, with straight chain aldehydes predominantly detected using headspace SPME. Hydrodistillation followed by liquid injection favoured compounds with higher boiling points, which is unsurprising given the high temperatures involved in the process and the potential loss of lower boiling compounds. These results highlighted that selection of an appropriate extraction technique is essential to detect and identify compounds that are present under ambient or physiological conditions.

### Transfer to the Analytical Instrument

#### Desorption, Focusing, and Injection Techniques

When sampling methods employ a sorbent or stationary phase for adsorption, two main approaches can be used to desorb the collected compounds; thermal desorption and solvent extraction (Ramírez et al. 2010; Stauffer et al. 2008; Wu and Chang 2013). Thermal desorption uses heat to volatilise compounds, releasing them from the sorbent and transferring them to the analytical instrument. In contrast, solvent extraction uses an appropriate solvent to elute collected compounds from the sorbent for subsequent analysis (Posudin 2014). Thermal desorption is often preferred because it avoids the use of extraction solvents and the associated solvent peak and dilution of analytes; however, it is primarily coupled with GC (Stauffer et al. 2008). While solvent extraction may result in solvent peaks that obscure analytes of interest, the resulting liquid extract can be stored, reanalysed, and used with alternative analytical techniques such as liquid chromatography.

To improve analytical sensitivity and chromatographic resolution, focusing techniques such as cryofocusing, can be used to pre-concentrate analytes before chromatographic separation (Weggler et al. 2020). During cryofocusing, analytes are trapped at low temperature in a cooled region or cryogenic trap and subsequently released as a narrow band by rapid heating for introduction onto the GC column (Weggler et al. 2020; Wilson et al. 2012). This pre-concentration produces narrower analyte bands, improving chromatographic peak shape and lowering detection limits. Programmed temperature vaporisation (PTV) and related pre-concentration and injection systems are also widely used to enhance the introduction of VOCs into GC, particularly for trace-level analytes or relatively large sample volumes (Terán-Baamonde et al. 2018; Yu et al. 2014; Zrostlíková et al. 2001). In PTV injection, the sample is introduced into a temperature-programmable inlet initially maintained at a relatively low temperature, allowing controlled solvent evaporation or venting while retaining the analytes of interest within the inlet. The inlet is then rapidly heated, transferring a concentrated band of analytes onto the GC column, thereby improving peak shape and lowering detection limits compared with conventional split/splitless injection. Similar principles underpin other large-volume and multi-step injection strategies that combine online pre-concentration with controlled thermal desorption. Such approaches can be integrated with sorbent-based sampling or headspace methods to maximise the transfer of VOCs into the analytical system while minimising solvent effects and matrix interferences.

### Detection and Identification

#### GC-Based Techniques

GC is one of the most commonly used techniques for separating volatile and semi-volatile compounds in complex mixtures, including those responsible for odours. Separation is governed by the distribution of compounds between the mobile gas phase and the stationary phase, which is influenced by their volatility and interactions with the stationary phase (Medeiros 2018). GC can be coupled with different detection modes, including flame ionisation detection (FID), mass spectrometry (MS), and olfactometry (O) (Wang and Jocelyn Paré 1997). The choice of detector typically depends on the nature of the sample and the intended analytical application.

##### GC–FID

The FID is one of the most widely used detectors for GC. It uses a hydrogen–air flame in which organic compounds eluting from the GC column are combusted, generating ions that produce an electrical signal approximately proportional to the amount of carbon entering the detector (Wang and Jocelyn Paré 1997). The resulting ions are collected between electrodes, generating an ion current that is measured as the detector response. GC–FID provides a sensitive and broadly applicable response for many organic compounds and is therefore well suited to the relative and quantitative measurement of compounds within mixtures. However, it does not provide structural information and therefore cannot, by itself, reliably identify unknown compounds (Bi et al. 2021). For these reasons, GC–FID is not suited to the identification of unknown components in complex mixtures, but it can be useful for quantifying previously identified compounds, for example when examining relationships between compound abundance and behavioural traits or temporal changes in chemical profiles. GC–FID is often used as a complementary technique alongside GC–MS and GC–O for quantification once compounds have been identified or confirmed, ideally using authentic analytical standards (Bi et al. 2021; Buchbauer et al. 1997).

##### GC–MS

**Fig. 2:**
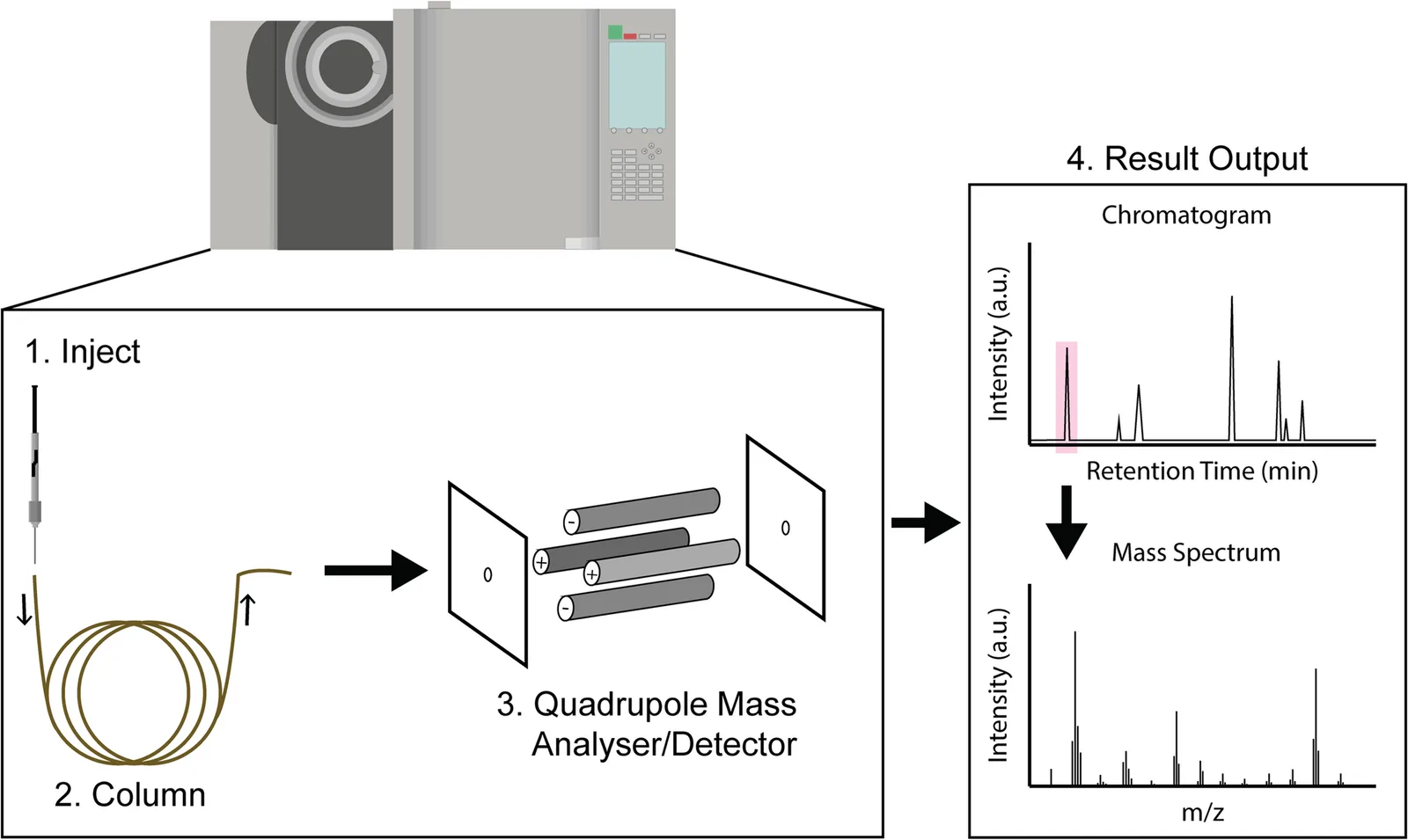
Schematic illustration of a gas chromatography instrument coupled to a mass spectrometer: (1) injection into the GC column; (2) separation of components on capillary column; (3) mass spectrometer detector; (4) dual output of chromatogram and mass spectrum for an individual component

GC–MS combines gas chromatographic separation with mass spectrometric detection to characterise individual components on the basis of their mass spectra, together with chromatographic retention information. Mass spectra can be compared with reference spectral libraries to provide tentative identifications, while comparison with authentic standards can provide more confident compound confirmation (Fig. 2). This technique therefore enables both qualitative and quantitative characterisation of VOCs and is one of the principal analytical techniques used for VOC profiling and identification. GC-MS is particularly useful for odour analysis when coupled with headspace sampling techniques (de Oliveira et al. 2008; Vas and Vékey 2004).

In GC, a sample is injected into a heated injection port where it is vaporised. The vapour is then carried through a capillary column by an inert carrier gas that serves as the mobile phase (Posudin 2014). Separation occurs within the column as compounds interact differently with the stationary phase coated on the inner wall of the capillary before reaching the detector, such as a mass spectrometer. In a mass spectrometer, neutral molecules are ionised and the resulting ions are separated according to their mass-to-charge ratios (*m/z*) by a mass analyser before detection. The resulting mass spectra provide structural information that can be used, together with chromatographic retention data and reference spectra or authentic standards, to characterise compounds separated by GC.

There are various sampling techniques suitable for GC–MS analysis, including liquid injection and headspace sampling. However, for VOC analysis, headspace techniques are commonly used because they preferentially sample compounds that partition into the gas phase while reducing the introduction of less-volatile matrix components.

GC–MS offers several important advantages, including high sensitivity, the ability to analyse small sample quantities, and the capability to characterise and tentatively identify unknown compounds, but it does not by itself establish which components contribute to perceived odour. Moreover, GC–MS is often limited by high initial instrument costs and on-going expenses associated with maintenance and specialised sampling equipment.

##### GC-O

GC-O is a technique that combines GC with human assessors as olfactory detectors (Delahunty et al. 2006; Friedrich and Acree 1998). By allowing assessors to evaluate individual compounds as they elute from the GC column, GC–O provides information on which components of a complex volatile mixture are odour-active and on their perceived odour characteristics (Brattoli et al. 2013). Odour-active compounds are not always the most abundant compounds in a sample. Therefore, GC–O can distinguish compounds that are odour-active from abundant compounds with little or no detectable odour (Brattoli et al. 2013; Egea et al. 2021). A complementary metric for evaluating the potential contribution of individual compounds to an overall odour is the odour activity value (OAV) calculated as the concentration of a compound divided by its odour threshold. An OAV > 1 indicates that the compound is present above its reported sensory threshold and may therefore contribute to the perceived odour, although interactions among compounds and variation in sensory thresholds mean that OAVs do not fully predict perception of a complex mixture (Egea et al. 2021). GC–O can also be combined with dilution-to-threshold approaches such as aroma extract dilution analysis (AEDA), in which an aroma extract is serially diluted and each dilution is analysed by GC–O. For each odour-active compound, the flavour dilution (FD) factor corresponds to the highest dilution at which the compound remains detectable by odour (Brattoli et al. 2013). There are three dilution methods that have been evaluated for HS–SPME–AEDA, (i) altering the amount of sample, (ii) diluting the sample, and (iii) changing the injection port split ratio (Feng et al. 2015). These approaches can help rank the relative sensory potency of compounds within a sample and identify compounds that remain perceptible at high dilution.

The addition of olfactometry as a detection method for GC complements instrumental detection by providing sensory information that cannot be obtained from GC–MS alone. GC-O can be combined with mass spectrometry (GC–MS/O or GC–O–MS), with the GC effluent split between the two detectors (Song and Liu 2018). GC–O–MS not only allows chemical characterisation of compounds by mass spectrometry, but also enables simultaneous sensory characterisation of their odours by human assessors. However, because olfactory sensitivity and perception can differ substantially among species, responses of human assessors should not be assumed to represent those of the target animal. Rather, GC–O provides a useful means of identifying compounds that are perceptible to humans and prioritising candidate odorants for subsequent behavioural or sensory testing in the species of interest.

#### General Considerations for the Analysis of VOCs Using GC

##### Ionisation Methods

Different ionisation methods in GC–MS offer complementary advantages for VOC analysis by providing different degrees of fragmentation, molecular-ion information, and sensitivity (Hejazi et al. 2009). Electron ionisation (EI) remains the most widely used ‘hard’ ionisation technique, producing extensive and highly reproducible fragmentation patterns that underpin most commercial libraries (e.g., NIST, Wiley), making it well suited to library-based non-target screening and structural characterisation of volatile and semi-volatile organic compounds. However, EI can produce a weak or absent molecular ion, which complicates molecular mass determination, particularly for compounds that undergo extensive fragmentation (Demarque et al. 2016). In such cases, softer ionisation methods such as chemical ionisation (CI) and field ionisation (FI) can complement EI by producing more prominent molecular or quasi-molecular ions with less fragmentation, thereby providing useful molecular-mass information. CI, using reagent gases such as methane or ammonia, can be operated in positive- or negative-ion modes, with negative chemical ionisation offering high selectivity and sensitivity for some electronegative compounds, including halogenated analytes, whereas FI generally produces abundant molecular ions with minimal fragmentation and can provide complementary molecular-mass information for compounds that fragment excessively under EI. In practice, VOC-focused GC–MS workflows typically use EI for routine library-based screening, while softer ionisation approaches, such as CI and, less commonly, FI, can provide additional molecular-ion information to support structural assignments.

##### Compound Identification

Compound identification in GC–MS relies on integrating chromatographic and mass spectrometric information, typically through a combination of fragmentation pattern interpretation, spectral library matching, and retention-based criteria. Under EI conditions, many VOCs produce characteristic and reproducible fragmentation patterns, which are compared against reference spectra in curated libraries (e.g., NIST) using similarity metrics. High-quality matches provide candidate or tentative identifications rather than definitive structural confirmation. Identification confidence can be increased by incorporating independent chromatographic information, particularly retention time or retention index (RI). Experimental RIs are commonly calculated relative to a homologous series of *n*-alkanes and compared with reference values obtained on the same or a comparable stationary phase (Kim et al. 2012; Wei et al. 2014). Library matching alone can yield incorrect assignments, particularly when candidate compounds have similar EI spectra, and retention information therefore provides an important complementary criterion for evaluating candidate identities (Kim and Zhang 2015; Wei et al. 2014). Where authentic standards are available, a higher level of confidence can be achieved by comparing both the mass spectrum and retention behaviour of the unknown with those of an authentic standard analysed under the same conditions.

##### Matrix Effects

Matrix effects are a critical consideration when analysing VOCs by headspace GC–MS because the sample matrix strongly influences analyte partitioning into the headspace, extraction efficiency, and ultimately the measured signal. For example, Insa et al. (2007) compared the HS–SPME analyses of chlorophenols in synthetic, white, and red wines and found significant differences between calibration slopes for different matrices, demonstrating that sample composition can substantially affect analytical response (Insa et al. 2007). Such matrix-dependent differences in analytical response can introduce quantitative bias when calibration standards do not adequately reproduce the matrix of the samples being analysed. Co-eluting matrix components may produce overlapping mass spectra or interfere with diagnostic ions, potentially compromising compound identification and quantification. A headspace GC–MS method developed for VOCs in reverse osmosis (RO) membranes demonstrated a substantial matrix effect and therefore employed VOC-free RO membrane material to construct matrix-matched calibration curves (Hou et al. 2022). This approach improved quantitative performance by exposing standards and samples to comparable matrix and headspace conditions.

Matrix effects can also arise from matrix-dependent partitioning and extraction during SPME. Pizarro et al. (2007) demonstrated that conventional single-extraction HS–SPME analysis of haloanisoles and volatile phenols in wines showed matrix-dependent analytical responses. They further showed that multiple headspace SPME involving successive extractions of the same sample, could reduce the influence of matrix-dependent partitioning by estimating the total analyte response from repeated extractions. These studies collectively emphasise that matrix effects in headspace GC–MS VOC analysis are often driven by the interplay between sample–headspace partitioning, sorbent–analyte interactions, and the composition of the sample matrix. For quantitative animal odour studies, these effects should be considered when comparing samples that differ substantially in matrix composition, and matrix-matched calibration or standard-addition approaches may be required where accurate quantification is a priority.

#### Mass Spectrometry-Based Techniques

##### PTR–MS

PTR–MS was developed for rapid, online detection and quantification of trace VOCs, utilising proton transfer reactions as a form of chemical ionisation prior to MS analysis (Blake et al. 2009). In PTR–MS, reagent ions, typically H_3_O^+^, transfer a proton to VOC molecules in a drift tube, generally when the proton affinity of the analyte exceeds that of water, resulting predominantly in protonated analyte ions. The pressure, electric field, and dimensions of the drift tube provide controlled conditions for ion–molecule reactions. H_3_O^+^ reagent ions are often produced using a hollow-cathode discharge with water vapor. The ion source and subsequent reactions with water are designed to produce H_3_O⁺ as the dominant reagent ion (Blake et al. 2009; Hansel et al. 1995). An electric field drives the ions through the drift tube, where the analyte is introduced and proton transfer reactions occur. The resulting protonated VOCs are transferred to a mass analyser and subsequently detected.

PTR–MS enables rapid, online VOC analysis through direct sampling without prior chromatographic separation, providing high temporal resolution. However, the absence of chromatographic separation can limit compound specificity because multiple compounds may contribute to the same *m/z*. This capability has been widely exploited in applications such as atmospheric VOC monitoring, where PTR–MS has been used for continuous measurement of biogenic and anthropogenic emissions (Romano and Hanna 2018), as well as in medical and clinical research (Herbig and Amann 2009), where PTR–MS and time-of-flight PTR–MS (PTR–TOF–MS) support real-time breath analysis for non-invasive assessment of metabolic status and potential disease-related biomarkers (Trefz et al. 2013). PTR–MS has also found extensive use in food and flavour science (Majchrzak et al. 2021) and plant physiology (Maleknia et al. 2007), for example in monitoring aroma release during food processing and characterising plant emissions under environmental stress. PTR–TOF–MS provides high mass resolving power and accurate-mass information, improving the ability to distinguish ions with similar nominal masses. Modern high-sensitivity instruments can achieve detection limits in the low-pptv or, under favourable conditions, sub-pptv range (Romano and Hanna 2018).

However, these advantages come with important limitations for compound idetification. Because PTR–MS often produces relatively simple mass spectra with limited fragmentation, different compounds can generate ions at the same nominal *m/z*, and structural isomers may generate indistinguishable or overlapping signals in the absence of chromatographic separation or complementary information, making unambiguous compound identification challenging. High-resolution TOF analysers can help resolve some nominally isobaric species by discriminating species with different exact masses and can in favourable cases support molecular-formula assignment. However, structural isomers generally require additional information. Strategies include the alternative reagent ions, particularly O_2_^+^, NO^+^, Kr^+^, or Xe^+^ in switcable-reagent-ion systems, tandem MS, or coupling PTR –MS with orthogonal separation techniques such as GC or ion mobility (Kaklamanos et al. 2016; Kohl et al. 2013). Consequently, PTR–MS is often best suited to real-time monitoring of VOC signals, temporal profiling, pattern-based classification, and the measurement of previously characterised marker compounds, whereas detailed structural characterisation and confident identification of unknown VOCs generally require complementary analytical information, for example from GC–MS or GC-coupled PTR–MS. The main practical limitations of PTR–MS include the relatively high cost and complexity of the instrumentation and the expertise required for data interpretation (Mazzucotelli et al. 2022).

##### SIFT–MS

Similar to PTR–MS, SIFT–MS is a soft chemical ionisation technique that typically enables direct, real-time analysis of complex VOC mixtures without prior chromatographic separation (Španěl and Smith 2020). SIFT–MS differs from PTR–MS in that its reagent ions (e.g., H_3_O^+^, NO^+^, O_2_^+^) generated by electrical discharge and are mass-selected before being introduced into a well-characterised flow tube, where they react with VOCs under controlled conditions (Blake et al. 2009; Langford et al. 2023; Španěl and Smith 2020). SIFT–MS allows for rapid switching between reagent ions, with switching between ions of different *m/z* possible on millisecond timescales. This enables multiple ion–molecule reaction chemistries to be examined within a single measurement sequence and can provide additional information for discriminating compounds, including some isomers (Langford et al. 2023). In addition to proton transfer, reactions between VOCs and the different reagent ions can proceed through pathways including charge transfer, hydride abstraction, and adduct formation, providing complementary product-ion patterns that can aid targeted compound discrimination and quantification (Langford et al. 2023). The reagent ions and analyte molecules traverse the flow tube under well-defined conditions, allowing analyte concentrations to be calculated from reagent- and product-ion count rates using known ion-molecule reaction rate coefficients and reaction times, which are defined by the controlled pressure, temperature and flow conditions within the flow tube (Španěl and Smith 2020).

Because PTR–MS and SIFT–MS typically analyse gas samples directly without prior chromatographic separation, real time odour analysis is possible, making these techniques particularly useful for environmental odour monitoring during animal production and other dynamic emission scenarios (Hansen et al. 2012). Although PTR–MS and SIFT–MS require specialised instrumentation with substantial acquisition and maintenance costs, their rapid, direct analysis can greatly increase sample throughput and reduce analysis time per sample compared with conventional chromatographic workflows (Majchrzak et al. 2018b; Perkins et al. 2023). The relative advantages of PTR–MS and SIFT–MS depend on the analytical application and instrument configuration. PTR–MS, particularly modern PTR–TOF–MS systems, can provide very high sensitivity and temporal resolution, whereas SIFT–MS offers rapid access to multiple reagent-ion chemistries and is particularly well suited to targeted, real-time quantification of VOCs. The use of multiple reagent ions can also provide additional selectivity in chemically complex mixtures, although unambiguous identification of isomeric compounds may still require complementary analytical information (Španěl and Smith 2020).

#### Electronic Nose

**Fig. 3:**
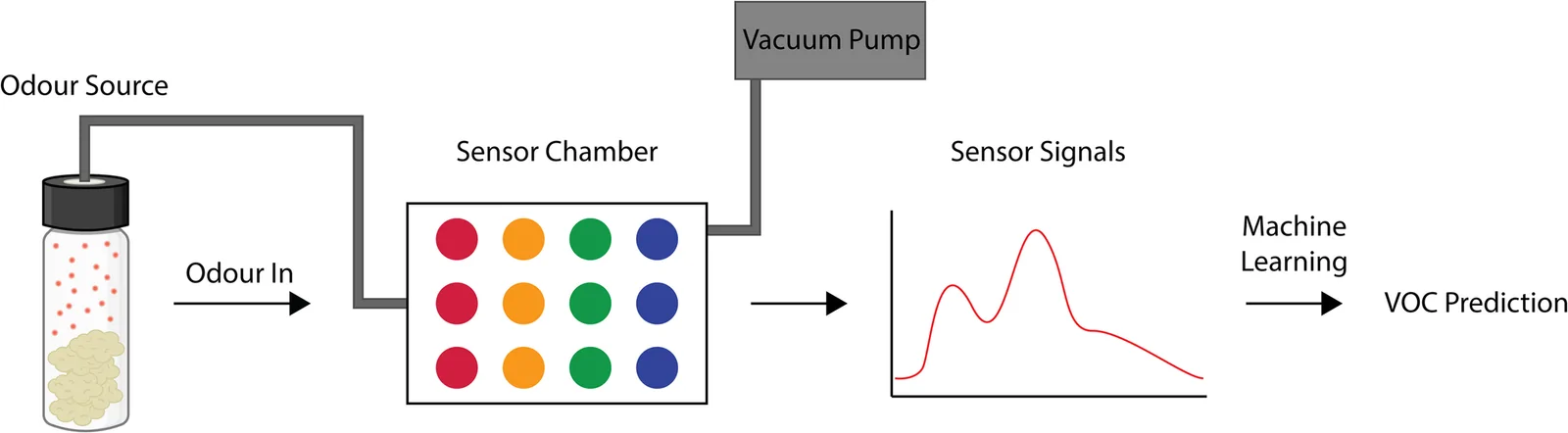
General schematic of an electronic nose for odour detection. (Adapted from (Tang et al. 2010)

The electronic nose was originally defined by Gardner and Bartlett (1994) as “an instrument which comprises an array of electronic chemical sensors with partial specificity, and an appropriate pattern-recognition system, capable of recognising simple or complex odours” (Gardner and Bartlett 1994). In recent years, electronic noses have gained increasing attention as rapid tools for detecting and classifying odour patterns, including in field applications. Electronic noses can be trained to classify new samples according to predefined odour classes. In environmental odour monitoring, dynamic olfactometry can also be used during training or calibration to characterise odour concentration and relate instrumental responses to human sensory perception (Bax et al. 2020; Brattoli et al. 2011). An electronic nose usually consists of a sample-delivery system, a detection chamber containing an array of partially selective sensors, and a data acquisition and pattern-recognition system (Fig. 3) (Corcoran 1993; Dymerski et al. 2011; Majchrzak et al. 2018a). The most common sensor types include chemiresistive sensors, such as metal oxide semiconductors or conducting polymers, which detect gases through changes in electrical resistance or conductance (Ameer and Adeloju 2005; James et al. 2005; Meixner and Lampe 1996). Other types of sensors include piezoelectric sensors, such as surface acoustic wave sensors and quartz crystal microbalances, which respond to analyte adsorption through changes in mass loading and oscillation frequency (Capelli et al. 2014; Fernández et al. 2007; Lieberzeit et al. 2008). Individually, these sensors are generally only partially selective and often respond to multiple compounds. However, differences in their cross-reactivity generate characteristic response patterns that can be used to discriminate among odour samples (Epping and Koch 2023; Wilson and Baietto 2009). During operation, air or sample headspace is delivered into a chemically inert chamber containing the sensor array (Fig. 3) (Bax et al. 2020). VOCs interact with the sensors, generating a multidimensional response pattern that is processed using statistical pattern-recognition or machine-learning algorithms to classify samples or predict properties for which the system has been calibrated, such as odour concentration.

A significant limitation of conventional electronic noses is their dependence on training data. Supervised classification models generally perform best for odour classes and conditions represented during training and may perform poorly when presented with substantially different samples. Importantly, although an electronic nose can classify a previously unmeasured sample according to learned response patterns, it does not normally identify the individual chemical constituents responsible for that pattern or elucidate the composition of a complex animal odour (Wysocka and Dębowski 2025). In addition, many electronic nose sensor types (particularly metal oxide and conducting polymer sensors) are highly sensitive to ambient humidity, which can alter baseline responses, reduce reproducibility, and necessitate humidity compensation, recalibration, or controlled measurement conditions (Ionescu et al. 2000). Cross-reactivity among sensors is fundamental to pattern-based discrimination but can also complicate interpretation, as each sensor often responds to multiple chemically diverse analytes, leading to overlapping response patterns and potential misclassification if the system is not carefully calibrated and validated for the specific odour matrix and operating conditions (Fitzgerald and Fenniri 2016). Long-term performance can also be affected by sensor poisoning and drift. Sensor poisoning occurs when strongly adsorbing or reactive contaminants alter the sensor surface, potentially causing persistent loss or alteration of sensor response, whereas sensor drift refers more broadly to gradual changes in sensor response over time caused by factors such as ageing, contamination, and environmental variation. Drift can be mitigated through approaches such as periodic calibration, reference-gas measurements, signal normalisation, and computational drift compensation (Pérez Pavón et al. 2006).

More recently, alternative instrument-based electronic nose approaches have employed rapid gas chromatography or mass spectrometry in combination with multivariate pattern recognition (Dymerski et al. 2011; Majchrzak et al. 2018a). In GC-based electronic noses, rapid chromatographic separation generates characteristic VOC fingerprints, with chromatographic peaks or retention-time regions serving as variables for subsequent pattern recognition (Dymerski et al. 2011). GC-based electronic noses can avoid some limitations associated with conventional chemical-sensor arrays, such as sensor poisoning, although instrumental signal stability and calibration remain important considerations (Dymerski et al. 2011; Pérez Pavón et al. 2006). In MS-based electronic noses, volatile samples can be introduced directly into the mass spectrometer without prior chromatographic separation (Majchrzak et al. 2018a; Martí et al. 2005). The intensities of selected ions at different *m/z* values can be treated as “pseudo-sensors”, collectively generating a mass-spectral fingerprint that can be analysed by multivariate methods (Martí et al. 2005). Although the mass-spectral data may provide some information about sample chemistry, the primary aim of an MS-based electronic nose is generally rapid pattern recognition and sample classification rather than comprehensive identification of individual VOCs. Beyond these instrument-based approaches, many conventional sensor-array electronic noses can be miniaturised, enabling portable and convenient field deployment (Baniţă et al. 2023).

Electronic noses have been investigated for disease detection in plants, animals and humans because they can detect disease-associated changes in VOC profiles, often using non-invasive samples (Wilson 2018). They have also been used in environmental odour monitoring, such as real-time estimation of odour concentrations from wastewater treatment plants (Burgués et al. 2021).

## Conclusions

Given the important role of olfaction in complex animal behaviours and interactions, characterising the VOCs underlying these scent cues is important for understanding and potentially manipulating animal responses. Several analytical approaches are available for characterising VOCs in animal-derived samples, each with its own advantages and limitations. Some methods are more sensitive to certain classes of compounds; for example, the performance of an electronic nose depends strongly on the type of sensors employed and the training data used to build classification models. Other analytical methods, such as GC–MS, can be tailored to detect multiple compound classes through the choice of column, ionisation mode, and sample preparation and extraction methods. Critically, alongside the choice of a particular analytical technique, the approach to sample collection and preparation can be equally important for obtaining meaningful results when analysing natural odours. Solvent extraction can recover less-volatile and matrix-associated compounds that may be poorly represented by gas-phase sampling, whereas distillation-based methods, such as hydrodistillation, preferentially recover compounds that withstand heating and distillation and may alter the original chemical profile through thermal degradation or transformation. In contrast, headspace-based sampling techniques generally provide a closer representation of volatile compounds released into the gas phase under defined environmental conditions, but can be biased towards more volatile compounds and are highly sensitive to experimental variables such as headspace volume, sorbent properties, and equilibration, extraction, and desorption conditions. Ultimately, no single sampling or analytical technique is universally optimal; therefore, the choice of methods, and where appropriate their combination, should be guided by the biological question, the chemical properties and volatility of the compounds of interest and the specific behavioural or ecological context in which the odour information will be interpreted. 

## Data Availability

No datasets were generated or analysed during the current study.

## References

[CR1] Abeel SM, Vickers AK, Decker D (1994) Trends in Purge and Trap. J Chromatogr Sci 32:328–338. 10.1093/chromsci/32.8.328

[CR2] Ameer Q, Adeloju SB (2005) Polypyrrole-based electronic noses for environmental and industrial analysis. Sens Actuators B 106:541–552. 10.1016/j.snb.2004.07.033

[CR3] Andrewartha T, Evans M, Blencowe A, Brewer K, Gordon IJ, Manning AD (2023) Landscapes of nausea: Successful conditioned taste aversion in a wild red fox population Conservation. Sci Pract 5:e12984. 10.1111/csp2.12984

[CR4] Apfelbach R, Blanchard CD, Blanchard RJ, Hayes RA, McGregor IS (2005) The effects of predator odors in mammalian prey species: A review of field and laboratory studies. Neurosci Biobehavioral Reviews 29:1123–1144. 10.1016/j.neubiorev.2005.05.005 16085312

[CR5] Baniţă C, Antoce OA, Cojocaru GA (2023) Evaluation by a GC Electronic Nose of the Differences in Volatile Profile Induced by Stopping Fermentation with Octanoic and Decanoic Acid to Produce. Sweet Wines Chemosensors 11:98. 10.3390/chemosensors11020098

[CR6] Banks PB, Bytheway JP, Carthey AJR, Hughes NK, Price CJ (2014) Olfaction and predator-prey interactions amongst mammals in Australia. In: Glen A, Dickman C (eds) Carnivores of Australia: Past, present and future, 1 edn. CSIRO Publishing, pp 405–415. doi:10.1071/978064310317720.389.404.2014.20

[CR7] Banks PB, Daly A, Bytheway JP (2016) Predator odours attract other predators. creating olfactory web Inform Biology Lett 12:20151053. 10.1098/rsbl.2015.1053 PMC4892236 27194283

[CR8] Bargmann CI (1997) Olfactory Receptors, Vomeronasal Receptors, and the Organization of Olfactory. Inform Cell 90:585–587. 10.1016/S0092-8674(00)80518-8 9288738

[CR9] Bax C, Sironi S, Capelli L (2020) How Can Odors Be Measured? An Overview of Methods and. Their Appl Atmos 11:92. 10.3390/atmos11010092

[CR10] Bi C et al (2021) Coupling a gas chromatograph simultaneously to a flame ionization detector and chemical ionization mass spectrometer for isomer-resolved measurements of particle-phase organic compounds. Atmos Meas Tech 14:3895–3907. 10.5194/amt-14-3895-2021

[CR11] Blake RS, Monks PS, Ellis AM (2009) Proton-Transfer Reaction Mass Spectrom Chem Reviews 109:861–896. 10.1021/cr800364q 19215144

[CR12] Brattoli M, De Gennaro G, De Pinto V, Demarinis Loiotile A, Lovascio S, Penza M (2011) Odour Detect Methods: Olfactometry Chem Sens Sens 11:5290–5322. 10.3390/s110505290 PMC3231359 22163901

[CR13] Brattoli M et al (2013) Gas Chromatography Analysis with Olfactometric Detection (GC-O) as a Useful Methodology for Chemical Characterization of. Odorous Compd Sens 13:16759–16800. 10.3390/s131216759 PMC3892869 24316571

[CR14] Brown WL Jr., Eisner T, Whittaker RH (1970) Allomones and Kairomones. Transspecific Chem Messengers BioScience 20:21–21. 10.2307/1294753

[CR15] Bruno T, Harries M (2019) Headspace Analysis: Purge and Trap. Encyclopedia of Analytical Science

[CR16] Buchbauer G, Jirovetz L, Nikiforov A (1997) Use of GC-FID, GC-FTIR-MS, and Olfactory Characterisation in the Analysis of Essential Oils and Plant Extracts. In: Linskens HF, Jackson JF (eds) Plant Volatile Analysis. Springer, Berlin Heidelberg, Berlin, Heidelberg, pp 97–117. doi:10.1007/978-3-662-03331-9_5

[CR17] Burger BV, Marx B, le Roux M, Burger WJG (2006) Simplified analysis of organic compounds in headspace and aqueous samples by high-capacity sample enrichment probe. J Chromatogr A 1121:259–267. 10.1016/j.chroma.2006.04.007 16696988

[CR18] Burger BV, Viviers MZ, le Roux NJ, Morris J, Bekker JPI, Le Roux M (2011) Olfactory Cue Mediated Neonatal Recognition in Sheep, Ovis aries. J Chem Ecol 37:1150–1163. 10.1007/s10886-011-0020-7 21948242

[CR19] Burger BV, Viviers MZ, le Roux NJ, Morris J, Bekker JPI, le Roux M (2013) Neonatal Recognition in Sheep. In: East ML, Dehnhard M (eds) Chemical Signals in Vertebrates 12. Springer New York, New York, NY, pp 61–75. doi:10.1007/978-1-4614-5927-9_5

[CR20] Burgués J, Esclapez MD, Doñate S, Marco S (2021) RHINOS: A lightweight portable electronic nose for real-time odor quantification in wastewater treatment plants iScience. 24:103371. 10.1016/j.isci.2021.103371 PMC8710464 34988386

[CR21] Bytheway JP, Price CJ, Banks PB (2016) Deadly intentions: naïve introduced foxes show rapid attraction to odour cues of an unfamiliar native prey. Sci Rep 6:30078. 10.1038/srep30078 PMC4945945 27416966

[CR22] Cadwallader K (2007) 18 - Measuring cheese flavor. In: Weimer BC (ed) Improving the Flavour of Cheese. Woodhead Publishing, pp 401–417. 10.1533/9781845693053.3.401

[CR23] Capelli L, Sironi S, Del Rosso R (2014) Electronic Noses for Environmental. Monit Appl Sens 14:19979–20007. 10.3390/s141119979 PMC4279467 25347583

[CR24] Cavaliere RM, Silvotti L, Percudani R, Tirindelli R (2020) Female mouse tears contain an anti-aggression pheromone. Sci Rep 10:2510. 10.1038/s41598-020-59293-9 PMC7018997 32054888

[CR25] Cavalli J-F, Fernandez X, Lizzani-Cuvelier L, Loiseau A-M (2003) Comparison of Static Headspace, Headspace Solid Phase Microextraction, Headspace Sorptive Extraction, and Direct Thermal Desorption Techniques on Chemical Composition of French Olive Oils. J Agric Food Chem 51:7709–7716. 10.1021/jf034834n 14664533

[CR26] Corcoran P (1993) Electronic odour sensing systems Electronics. Communication Eng J 5:303–308. 10.1049/ecej:19930060

[CR27] Cork A, Hall MJR (2007) Development of an odour-baited target for female New World screwworm, Cochliomyia hominivorax: studies with host baits and synthetic wound fluids. Med Vet Entomol 21:85–92. 10.1111/j.1365-2915.2006.00661.x 17373950

[CR28] Cowan DP, Reynolds JC, Gill EL (2000) Reducing predation through conditioned taste aversion. In: Gosling LM, Sutherland WJ (eds) Behaviour and Conservation. Cambridge University Press, pp 281–299

[CR29] de Oliveira LS et al (2008) Headspace solid phase microextraction/gas chromatography–mass spectrometry combined to chemometric analysis for volatile organic compounds determination in canine hair: A new tool to detect dog contamination by visceral leishmaniasis. J Chromatogr B 875:392–398. 10.1016/j.jchromb.2008.09.028 18945650

[CR30] Delahunty CM, Eyres G, Dufour J-P (2006) Gas chromatography-olfactometry. J Sep Sci 29:2107–2125. 10.1002/jssc.200500509 17069240

[CR31] Demarque DP, Crotti AEM, Vessecchi R, Lopes JLC, Lopes NP (2016) Fragmentation reactions using electrospray ionization mass spectrometry: an important tool for the structural elucidation and characterization of synthetic and natural products. Nat Prod Rep 33:432–455. 10.1039/c5np00073d 26673733

[CR32] Demeestere K, Dewulf J, De Witte B, Van Langenhove H (2007) Sample preparation for the analysis of volatile organic compounds in air and water matrices. J Chromatogr A 1153:130–144. 10.1016/j.chroma.2007.01.012 17258752

[CR33] Deshpande K, Furton KG, Mills DEK (2018) The Equine Volatilome: Volatile Organic Compounds as Discriminatory Markers. J Equine Veterinary Sci 62:47–53. 10.1016/j.jevs.2017.05.013

[CR34] Dickman CR, Doncaster CP (1984) Responses of small mammals to Red fox (Vulpes vulpes) odour. J Zool 204:521–531. 10.1111/j.1469-7998.1984.tb02384.x

[CR35] Doty RL (1986) Odor-guided behavior. Mamm Experientia 42:257–271. 10.1007/BF01942506 3514263

[CR36] Dulac C, Torello AT (2003) Molecular detection of pheromone signals in mammals: from genes to behaviour. Nat Rev Neurosci 4:551–562. 10.1038/nrn1140 12838330

[CR37] Dymerski TM, Chmiel TM, Wardencki W (2011) Invited Review Article: An odor-sensing system—powerful technique for foodstuff studies. Rev Sci Instrum 82:111101. 10.1063/1.3660805 22128959

[CR38] Egea MB, Bertolo MR, Oliveira Filho JG, Lemes AC (2021) A Narrative Review of the Current Knowledge on Fruit Active Aroma Using Gas Chromatography-Olfactometry (GC-O) Analysis. Molecules 26:5181. 10.3390/molecules26175181 PMC8433627 34500614

[CR39] Epping R, Koch M (2023) On-Site Detection of Volatile Organic Compounds (VOCs). Molecules 28:1598. 10.3390/molecules28041598 PMC9966347 36838585

[CR40] Feng Y, Cai Y, Sun-Waterhouse D, Cui C, Su G, Lin L, Zhao M (2015) Approaches of aroma extraction dilution analysis (AEDA) for headspace solid phase microextraction and gas chromatography–olfactometry (HS-SPME–GC–O): Altering sample amount, diluting the sample or adjusting split ratio? Food Chem 187:44–52. 10.1016/j.foodchem.2015.03.138 25976996

[CR41] Fernández MJ et al (2007) Discrimination of volatile compounds through an electronic nose based on ZnO SAW sensors. Sens Actuators B 127:277–283. 10.1016/j.snb.2007.07.054

[CR42] Finnerty PB, McArthur C, Banks P, Price C, Shrader AM (2022) The Olfactory Landscape Concept: A Key Source of Past, Present, and Future Information Driving Animal Movement and Decision-making BioScience. 72:745–752. 10.1093/biosci/biac039 PMC9343233 35923186

[CR43] Finnerty PB, Possell M, Banks PB, Orlando CG, Price CJ, Shrader AM, McArthur C (2024) Olfactory misinformation provides refuge to palatable plants from mammalian browsing. Nat Ecol Evol 8:645–650. 10.1038/s41559-024-02330-x PMC11009103 38307993

[CR44] Fitzgerald JE, Fenniri H (2016) Biomimetic cross-reactive sensor arrays. prospects biodiagnostics RSC Adv 6:80468–80484. 10.1039/c6ra16403j PMC5312755 28217300

[CR45] Flórez Menéndez JC, Fernández Sánchez ML, Fernández Martínez E, Sánchez Uría JE, Sanz-Medel A (2004) Static headspace versus head space solid-phase microextraction (HS-SPME) for the determination of volatile organochlorine compounds in landfill leachates by. gas Chromatogr Talanta 63:809–814. 10.1016/j.talanta.2003.12.044 18969504

[CR46] Foy MR, Foy JG (2009) Conditioned Taste Aversion. In: Squire LR (ed) Encyclopedia of Neuroscience. Academic, Oxford, pp 35–41. doi:10.1016/B978-008045046-9.00112-1

[CR48] Frank DF, Erb HN, Houpt KA (1999) Urine spraying in cats: presence of concurrent disease and effects of a pheromone treatment Applied. Anim Behav Sci 61:263–272. 10.1016/S0168-1591(98)00195-6

[CR47] Frank D, Beauchamp G, Palestrini C (2010) Systematic review of the use of pheromones for treatment of undesirable behavior in cats and dogs. J Am Vet Med Assoc 236:1308–1316. 10.2460/javma.236.12.1308 20550445

[CR49] Friedrich JE, Acree TE (1998) Gas Chromatography Olfactometry (GC/O) of Dairy Products. Int Dairy J 8:235–241. 10.1016/S0958-6946(98)80002-2

[CR50] Gardner JW, Bartlett PN (1994) A brief history of electronic noses. Sens Actuators B 18:210–211. 10.1016/0925-4005(94)87085-3

[CR51] Garvey PM, Glen AS, Pech RP (2016) Dominant predator odour triggers caution and eavesdropping behaviour in a mammalian mesopredator. Behav Ecol Sociobiol 70:481–492. 10.1007/s00265-016-2063-9

[CR52] Griffith CA, Steigerwald ES, Buffington CAT (2000) Effects of a synthetic facial pheromone on behavior of cats. J Am Vet Med Assoc 217:1154–1156. 10.2460/javma.2000.217.1154 11043684

[CR53] Gunn-Moore DA, Cameron ME (2004) A pilot study using synthetic feline facial pheromone for the management of feline idiopathic cystitis. J Feline Med Surg 6:133–138. 10.1016/j.jfms.2004.01.006 PMC10822346 15135349

[CR54] Gustavson CR, Garcia J, Hankins WG, Rusiniak KW (1974) Coyote Predation Control Aversive Conditioning Sci 184:581–583. 10.1126/science.184.4136.581 17755036

[CR55] Gustavson CR, Gustavson JC, Holzer GA (1983) Thiabendazole-based taste aversions in dingoes (*Canis familiaris dingo*) and New Guinea wild dogs (*Canis familiaris hallstromi*). Appl Anim Ethol 10:385–388. 10.1016/0304-3762(83)90187-6

[CR56] Hansel A, Jordan A, Holzinger R, Prazeller P, Vogel W, Lindinger W (1995) Proton transfer reaction mass spectrometry: on-line trace gas analysis at the ppb level International. J Mass Spectrom Ion Processes 149–150:609–619. 10.1016/0168-1176(95)04294-U

[CR57] Hansen MJ, Liu D, Guldberg LB, Feilberg A (2012) Application of Proton-Transfer-Reaction Mass Spectrometry to the Assessment of Odorant Removal in a Biological Air Cleaner for Pig Production. J Agric Food Chem 60:2599–2606. 10.1021/jf300182c 22304479

[CR58] Harrington LA, Harrington AL, Macdonald DW (2009) The Smell of New Competitors: The Response of American Mink, Mustela vison, to the Odours of Otter, Lutra lutra and Polecat. M putorius Ethol 115:421–428. 10.1111/j.1439-0310.2008.01593.x

[CR59] Hasbay I, Galanakis CM (2018) 7 - Recovery technologies and encapsulation techniques. In: Galanakis CM (ed) Polyphenols: Properties, Recovery, and Applications. Woodhead Publishing, pp 233–264. doi:10.1016/B978-0-12-813572-3.00007-5

[CR60] Haug H, Klein L, Sauerwald T, Poelke B, Beauchamp J, Roloff A (2024) Sampling Volatile Organic Compound Emissions from Consumer Products. Rev Crit Reviews Anal Chem 54:1895–1916. 10.1080/10408347.2022.2136484 36306209

[CR61] Hejazi L, Ebrahimi D, Hibbert DB, Guilhaus M (2009) Compatibility of electron ionization and soft ionization methods in gas chromatography/orthogonal time-of-flight mass spectrometry. Rapid Commun Mass Spectrom 23:2181–2189. 10.1002/rcm.4131 19530152

[CR62] Herbig J, Amann A (2009) Proton Transfer Reaction-Mass Spectrometry Applications in Medical Research. J Breath Res 3:020201. 10.1088/1752-7163/3/2/020201 21383455

[CR63] Holderman CJ, Kaufman PE, Booth MM, Bernier UR (2017) Novel collection method for volatile organic compounds (VOCs) from dogs. J Chromatogr B 1061–1062:1–4. 10.1016/j.jchromb.2017.06.044 28692852

[CR64] Hou M et al (2022) Development of a headspace-gas chromatography/mass spectrometry method based on matrix-matched calibration for evaluating VOC content, characterization, source, and risk in RO membrane. Polym Test 107:107474. 10.1016/j.polymertesting.2022.107474

[CR65] Hubbard DT, Blanchard DC, Yang M, Markham CM, Gervacio A, Chun-I L, Blanchard RJ (2004) Development of defensive behavior and conditioning to cat odor in the rat. Physiol Behav 80:525–530. 10.1016/j.physbeh.2003.10.006 14741237

[CR66] Hughes NK, Price CJ, Banks PB (2010) Predators Are Attracted to the Olfactory Signals of. Prey PLoS ONE 5:e13114. 10.1371/journal.pone.0013114 PMC2948037 20927352

[CR67] Hughes NK, Kelley JL, Banks PB (2012) Dangerous liaisons: the predation risks of receiving social signals. Ecol Lett 15:1326–1339. 10.1111/j.1461-0248.2012.01856.x 22925009

[CR68] Insa S, Besalú E, Salvadó V, Anticó E (2007) Assessment of the matrix effect on the headspace solid-phase microextraction (HS-SPME) analysis of chlorophenols in wines. J Sep Sci 30:722–730. 10.1002/jssc.200600021 17461113

[CR69] Ionescu R, Vancu A, Tomescu A (2000) Time-dependent humidity calibration for drift corrections in electronic noses equipped with SnO2 gas sensors. Sens Actuators B 69:283–286. 10.1016/S0925-4005(00)00508-6

[CR70] James D, Scott SM, Ali Z, O’Hare WT (2005) Chemical Sensors for Electronic. Nose Syst Microchimica Acta 149:1–17. 10.1007/s00604-004-0291-6

[CR71] Jeleń H, Gracka A, Myśków B (2017) Static Headspace Extraction with Compounds Trapping for the Analysis of Volatile Lipid Oxidation Products. Food Anal Methods 10:2729–2734. 10.1007/s12161-017-0838-x

[CR72] Jemiolo B, Harvey S, Novotny M (1986) Promotion of the Whitten effect in female mice by synthetic analogs of male urinary constituents. Proc Natl Acad Sci 83:4576–4579. 10.1073/pnas.83.12.4576 PMC323778 3459193

[CR73] Johansson A, Birgersson G, Schlyter F (2019) Using synthetic semiochemicals to train canines to detect bark beetle–infested trees. Ann For Sci 76:58. 10.1007/s13595-019-0841-z

[CR74] Jolly CJ, Kelly E, Gillespie GR, Phillips B, Webb JK (2017) Out of the frying pan: Reintroduction of toad-smart northern quolls to southern Kakadu National. Park Austral Ecol 43:139–149. 10.1111/aec.12551

[CR75] Kaklamanos G, Aprea E, Theodoridis G (2016) Mass Spectrometry: Principles and Instrumentation. In: Caballero B, Finglas PM, Toldrá F (eds) Encyclopedia of Food and Health. Academic, Oxford, pp 661–668. doi:10.1016/B978-0-12-384947-2.00447-5

[CR77] Kim S, Zhang X (2015) Discovery of false identification using similarity difference in GC–MS-based metabolomics. J Chemom 29:80–86. 10.1002/cem.2665 PMC4414261 25937705

[CR76] Kim S, Koo I, Jeong J, Wu S, Shi X, Zhang X (2012) Compound Identification Using Partial and Semipartial Correlations for Gas Chromatography–Mass. Spectrom Data Anal Chem 84:6477–6487. 10.1021/ac301350n PMC3418476 22794294

[CR78] Kohl I, Herbig J, Dunkl J, Hansel A, Daniaux M, Hubalek M (2013) Chap. 6 - Smokers Breath as Seen by Proton-Transfer-Reaction Time-of-Flight Mass Spectrometry (PTR-TOF-MS). In: Amann A, Smith D (eds) Volatile Biomarkers. Elsevier, Boston, pp 89–116. doi:10.1016/B978-0-44-462613-4.00006-4

[CR79] Kost C (2008) Chemical Communication. In: Jørgensen SE, Fath BD (eds) Encyclopedia of Ecology. Academic, Oxford, pp 557–575. doi:10.1016/B978-008045405-4.00036-7

[CR80] Kremser A, Jochmann MA, Schmidt TC (2016) Anal Bioanal Chem 408:6567–6579. 10.1007/s00216-016-9843-y. Systematic comparison of static and dynamic headspace sampling techniques for gas chromatography 27526093

[CR81] Landsberg GM, Beck A, Lopez A, Deniaud M, Araujo JA, Milgram NW (2015) Dog-appeasing pheromone collars reduce sound-induced fear and anxiety in beagle dogs: a placebo-controlled study. Vet Rec 177:260–260. 10.1136/vr.103172 PMC4602264 26311736

[CR82] Langford VS, Dryahina K, Španěl P (2023) Robust Automated SIFT-MS Quantitation of Volatile Compounds in Air Using a Multicomponent Gas Standard. J Am Soc Mass Spectrom 34:2630–2645. 10.1021/jasms.3c00312 PMC10704587 37988479

[CR83] Liberles SD (2014) Mammalian Pheromones Annual Rev Physiol 76:151–175. 10.1146/annurev-physiol-021113-170334 PMC4310675 23988175

[CR84] Lieberzeit PA, Rehman A, Najafi B, Dickert FL (2008) Real-life application of a QCM-based e-nose: quantitative characterization of different plant-degradation processes. Anal Bioanal Chem 391:2897–2903. 10.1007/s00216-008-2222-6 18581101

[CR85] Lisovac AM, Shooter D (2003) Volatiles from sheep wool and the modification of wool odour. Small Ruminant Res 49:115–124. 10.1016/S0921-4488(03)00075-0

[CR86] Majchrzak T, Wojnowski W, Dymerski T, Gębicki J, Namieśnik J (2018a) Electronic noses in classification and quality control of edible oils: A. Rev Food Chem 246:192–201. 10.1016/j.foodchem.2017.11.013 29291839

[CR87] Majchrzak T, Wojnowski W, Lubinska-Szczygeł M, Różańska A, Namieśnik J, Dymerski T (2018b) PTR-MS and GC-MS as complementary techniques for analysis of volatiles: A tutorial review. Anal Chim Acta 1035:1–13. 10.1016/j.aca.2018.06.056 30224127

[CR88] Majchrzak T, Wojnowski W, Wasik A (2021) Revealing dynamic changes of the volatile profile of food samples using. PTR–MS Food Chem 364:130404. 10.1016/j.foodchem.2021.130404 34175628

[CR89] Maleknia SD, Bell TL, Adams MA (2007) PTR-MS analysis of reference and plant-emitted volatile organic compounds International. J Mass Spectrom 262:203–210. 10.1016/j.ijms.2006.11.010

[CR90] Mallia S, Fernández-García E, Olivier Bosset J (2005) Comparison of purge and trap and solid phase microextraction techniques for studying the volatile aroma compounds of three European PDO hard cheeses. Int Dairy J 15:741–758. 10.1016/j.idairyj.2004.11.007

[CR91] Manning AD, Andrewartha TA, Blencowe A, Brewer K, Gordon IJ, Evans MJ (2021) Bettering the devil you know: Can we drive predator adaptation to restore native fauna? Conserv Sci Pract 3:e447. 10.1111/csp2.447

[CR92] Marsili RT (1999) Comparison of Solid-Phase Microextraction and Dynamic Headspace Methods for the Gas Chromatographic—Mass Spectrometric Analysis of Light-Induced Lipid Oxidation Products in Milk. J Chromatogr Sci 37:17–23. 10.1093/chromsci/37.1.17 9987853

[CR93] Martí MP, Busto O, Guasch J, Boqué R (2005) Electronic noses in the quality control of alcoholic beverages TrAC. Trends Anal Chem 24:57–66. 10.1016/j.trac.2004.09.006

[CR94] Mazzucotelli M et al (2022) Proton transfer reaction mass spectrometry: A green alternative for food volatilome profiling. Green Anal Chem 3:100041. 10.1016/j.greeac.2022.100041

[CR95] Medeiros PM (2018) Gas Chromatography–Mass Spectrometry (GC–MS). In: White WM (ed) Encyclopedia of Geochemistry: A Comprehensive Reference Source on the Chemistry of the Earth. Springer International Publishing, Cham, pp 530–535. doi:10.1007/978-3-319-39312-4_159

[CR96] Meixner H, Lampe U (1996) Metal oxide sensors. Sens Actuators B 33:198–202. 10.1016/0925-4005(96)80098-0

[CR97] Murphy EC et al (2022) Body Odours as Lures for Stoats Mustela erminea. Captive Field Trials Anim 12:394. 10.3390/ani12030394 PMC8833509 35158715

[CR98] Nielsen BL et al (2015) Olfaction: An Overlooked Sensory Modality in Applied Ethology and Animal Welfare. Front Veterinary Sci 2:69. 10.3389/fvets.2015.00069 PMC4672279 26664995

[CR99] Norbury GL et al (2021) Misinformation tactics protect rare birds from problem predators. Sci Adv 7:eabe4164. 10.1126/sciadv.abe4164 PMC7946364 33692107

[CR100] Novotny M, Harvey S, Jemiolo B, Alberts J (1985) Synthetic pheromones that promote inter-male aggression in mice. Proc Natl Acad Sci 82:2059–2061. 10.1073/pnas.82.7.2059 PMC397491 3856883

[CR101] Novotny MV, Ma W, Wiesler D, Zídek L (1999) Positive identification of the puberty-accelerating pheromone of the house mouse: the volatile ligands associating with the major urinary protein. Proc Royal Soc Lond Ser B: Biol Sci 266:2017–2022. 10.1098/rspb.1999.0880 PMC1690305 10584338

[CR102] Pageat P, Gaultier E (2003) Current research in canine and feline pheromones Veterinary Clinics of North America. Small Anim Pract 33:187–211. 10.1016/S0195-5616(02)00128-6 12701508

[CR103] Parker FCG, Price CJ, Bytheway JP, Banks PB (2023) Olfactory misinformation reduces wheat seed loss caused by rodent pests. Nat Sustain 6:1041–1044. 10.1038/s41893-023-01127-3

[CR139] Pizarro C, Pérez-del-Notario N, González-Sáiz JM (2007) Multiple headspace solid-phase microextraction for eliminating matrix effect in the simultaneous determination of haloanisoles and volatile phenols in wines. J Chromatogr A 1166(1-2):1–8 10.1016/j.chroma.2007.08.031 17727868

[CR104] Pérez Pavón JL, del Nogal Sánchez M, Pinto CG, Fernández Laespada ME, Cordero BM, Peña AG (2006) Strategies for qualitative and quantitative analyses with mass spectrometry-based electronic noses TrAC. Trends Anal Chem 25:257–266. 10.1016/j.trac.2005.09.003

[CR105] Perkins MJ, Hastie C, Whitlock SE, Langford VS (2023) Pharmaceutical Residual Solvent Analysis: A Comparison of GC-FID and SIFT-MS Performance. AppliedChem 3:290–302. 10.3390/appliedchem3020018

[CR106] Posudin Y (2014) Methods of Analysis of Volatile Organic Compounds. In: Posudin Y (ed) Methods of Measuring Environmental Parameters. pp 229–243. 10.1002/9781118914236.ch18

[CR107] Price C, McArthur C, Norbury G, Banks P (2022) Olfactory misinformation: creating fake news to reduce problem foraging by wildlife. Front Ecol Environ 20:531–538. 10.1002/fee.2534

[CR108] Ramírez N, Cuadras A, Rovira E, Borrull F, Marcé RM (2010) Comparative study of solvent extraction and thermal desorption methods for determining a wide range of volatile organic compounds in. ambient air Talanta 82:719–727. 10.1016/j.talanta.2010.05.038 20602960

[CR109] Romano A, Hanna GB (2018) Identification and quantification of VOCs by proton transfer reaction time of flight mass spectrometry: An experimental workflow for the optimization of specificity, sensitivity, and accuracy. J Mass Spectrom 53:287–295. 10.1002/jms.4063 PMC5838793 29336521

[CR110] Schlyter F, Birgersson G, Leufvén A (1989) Inhibition of attraction to aggregation pheromone by verbenone and ipsenol. J Chem Ecol 15:2263–2277. 10.1007/BF01014114 24272385

[CR111] Sih A et al (2010) Predator–prey naïveté, antipredator behavior, and the ecology of. Predat invasions Oikos 119:610–621. 10.1111/j.1600-0706.2009.18039.x

[CR112] Song H, Liu J (2018) GC-O-MS technique and its applications in food flavor analysis. Food Res Int 114:187–198. 10.1016/j.foodres.2018.07.037 30361015

[CR113] Španěl P, Smith D (2020) Quantification of volatile metabolites in exhaled breath by selected ion flow tube mass spectrometry. SIFT-MS Clin Mass Spectrom 16:18–24. 10.1016/j.clinms.2020.02.001 PMC8601014 34820516

[CR114] Stauffer E, Dolan JA, Newman R (2008) CHAPTER 11 - Extraction of Ignitable Liquid Residues from Fire Debris. In: Stauffer E, Dolan JA, Newman R (eds) Fire Debris Analysis. Academic, Burlington, pp 377–439. doi:10.1016/B978-012663971-1.50015-4

[CR115] Stowers L, Marton TF (2005) What Is a Pheromone? Mammalian Pheromones Reconsidered Neuron 46:699–702. 10.1016/j.neuron.2005.04.032 15924856

[CR116] Tang K-T, Chiu S-W, Pan C-H, Hsieh H-Y, Liang Y-S, Liu S-C (2010) Development of a Portable Electronic Nose System for the Detection and Classification of. Fruity Odors Sens 10:9179–9193. 10.3390/s101009179 PMC3230968 22163403

[CR117] Terán-Baamonde J, Bouchet S, Tessier E, Amouroux D (2018) Development of a large volume injection method using a programmed temperature vaporization injector – gas chromatography hyphenated to ICP-MS for the simultaneous determination of mercury, tin and lead species at ultra-trace levels in natural waters. J Chromatogr A 1547:77–85. 10.1016/j.chroma.2018.02.056 29555356

[CR118] Tobajas J, Gómez-Ramírez P, María-Mojica P, Navas I, García-Fernández AJ, Ferreras P, Mateo R (2019) Conditioned food aversion mediated by odour cue and microencapsulated levamisole to avoid predation by canids. Eur J Wildl Res 65:32. 10.1007/s10344-019-1271-9

[CR119] Tobajas J, Descalzo E, Villafuerte R, Jimenez J, Mateo R, Ferreras P (2021) Conditioned odor aversion as a tool for reducing post-release predation during animal translocations. Anim Conserv 24:373–385. 10.1111/acv.12643

[CR120] Tod E, Brander D, Waran N (2005) Efficacy of dog appeasing pheromone in reducing stress and fear related behaviour in shelter dogs. Appl Anim Behav Sci 93:295–308. 10.1016/j.applanim.2005.01.007

[CR121] Trefz P et al (2013) Continuous Real Time Breath Gas Monitoring in the. -Mass Spectrom Anal Chem 85:10321–10329. 10.1021/ac402298v. Clinical Environment by Proton-Transfer-Reaction-Time-of-Flight 24044609

[CR122] Tsikolia M et al (2022) Studies on the Volatiles Composition of Stored Sheep Wool, and Attractancy toward Aedes. aegypti Mosquitoes Insects 13:208. 10.3390/insects13020208 PMC8879698 35206782

[CR123] van Hout MWJ, Niederländer HAG, de Zeeuw RA, de Jong GJ (2003) Chap. 1 - New developments in integrated sample preparation for bioanalysis. In: Wilson ID (ed) Handbook of Analytical Separations, vol 4. Elsevier Science B.V., pp 1–44. 10.1016/S1567-7192(03)80002-5

[CR124] Vas G, Vékey K (2004) Solid-phase microextraction: a powerful sample preparation tool prior to mass spectrometric analysis. J Mass Spectrom 39:233–254. 10.1002/jms.606 15039931

[CR125] Vyviurska O, Hanobiková M, Gomes AA, Špánik I (2021) Multivariate optimization of dual-sorbent dynamic headspace extraction of volatiles in wine analysis. Food Chem 365:130449. 10.1016/j.foodchem.2021.130449 34218105

[CR126] Walker D, Vaglio S (2021) Sampling and Analysis of Animal Scent. Signals J Vis Exp:e60902. 10.3791/60902 33645562

[CR127] Wang Z, Jocelyn Paré JR (1997) Chap. 3 Gas chromatography (GC): Principles and applications. In: Paré JRJ, Bélanger JMR (eds) Techniques and Instrumentation in Analytical Chemistry, vol 18. Elsevier, pp 61–91. doi:10.1016/S0167-9244(97)80012-1

[CR129] Ward JF, Macdonald DW, Doncaster CP (1997) Responses of foraging hedgehogs to badger odour. Anim Behav 53:709–720. 10.1006/anbe.1996.0307

[CR128] Ward-Fear G, Thomas J, Webb JK, Pearson DJ, Shine R (2017) Eliciting conditioned taste aversion in lizards: live toxic prey are more effective than scent and taste cues alone. Integr Zool 12:112–120. 10.1111/1749-4877.12226 27579495

[CR130] Weggler BA, Gruber B, Teehan P, Jaramillo R, Dorman FL (2020) Chap. 5 - Inlets and sampling. In: Snow NH (ed) Separation Science and Technology, vol 12. Academic Press, pp 141–203. 10.1016/B978-0-12-813745-1.00005-2

[CR131] Wei X, Koo I, Kim S, Zhang X (2014) Compound identification in GC-MS by simultaneously evaluating the mass spectrum and. Retent index Analyst 139:2507–2514. 10.1039/c3an02171h PMC4308046 24665464

[CR133] Wilson AD (2018) Applications of Electronic-Nose Technologies for Noninvasive Early Detection of Plant. Anim Hum Dis Chemosensors 6:45. 10.3390/chemosensors6040045

[CR132] Wilson AD, Baietto M (2009) Applications and Advances in Electronic-Nose. Technol Sens 9:5099–5148. 10.3390/s90705099 PMC3274163 22346690

[CR134] Wilson RB, Fitz BD, Mannion BC, Lai T, Olund RK, Hoggard JC, Synovec RE (2012) High-speed cryo-focusing injection for gas chromatography: Reduction of injection band broadening with. concentration Enrich Talanta 97:9–15. 10.1016/j.talanta.2012.03.054 22841041

[CR135] Wu Y, Chang VWC (2013) Comparison of solvent extraction and thermal desorption methods for determination of volatile polyfluorinated alkyl substances in the urban atmosphere. Anal Methods 5:3410–3417. 10.1039/C3AY40089A

[CR136] Wysocka I, Dębowski M (2025) Advantages and Limitations of Measurement Methods for Assessing Odour Nuisance in Air—A Comparative Review. Appl Sci 15:5622. 10.3390/app15105622

[CR137] Yu B, Song Y, Yu H, Han L, Liu H (2014) Optimizations of large volume-direct aqueous injection-gas chromatography to monitor volatile organic compounds in surface water. Anal Methods 6:6931–6938. 10.1039/c4ay00266k 24997514

[CR138] Zrostlíková J, Hajšlová J, Godula M, Maštovská K (2001) Performance of programmed temperature vaporizer, pulsed splitless and on-column injection techniques in analysis of pesticide residues in plant matrices. J Chromatogr A 937:73–86. 10.1016/S0021-9673(01)01308-5 11765087

